# Prognostic indicators in peritoneal carcinomatosis from gastrointestinal cancer

**DOI:** 10.1186/1477-7800-2-3

**Published:** 2005-02-08

**Authors:** Rhonda L Harmon, Paul H Sugarbaker

**Affiliations:** 1 Washington Cancer Institute, Washington Hospital Center, 110 Irving St., NW, Washington, DC, 20010 USA

**Keywords:** Carcinomatosis, prognostic indicators, peritonectomy, cytoreductive surgery, colorectal cancer, gastric cancer, intraperitoneal chemotherapy.

## Abstract

Peritoneal carcinomatosis from gastrointestinal cancer has new treatment options for surgical management. The approach uses cytoreductive surgery which combines peritonectomy and visceral resection in an effort to remove all visible cancer within the abdomen and pelvis. Then the peritoneal cavity is flooded with chemotherapy solution in an attempt to eradicate residual disease. In order to select patients for this approach the quantitative prognostic indicators for carcinomatosis were reviewed, compared and contrasted. Prognostic indicators to be used to select patients for this aggressive approach at the initiation of surgery and after completion of cytoreduction were studied. Four quantitative assessments to be used at the time of abdominal exploration were the Gilly staging, Japanese gastric cancer P score, peritoneal cancer index (PCI), and the simplified peritoneal cancer index (SPCI). All have value with the PCI being the most validated and most precise. Preoperative assessments include the tumor histopathology and the prior surgical score. The completeness of cytoreduction score is an assessment of residual disease after a maximal surgical effort. An opportunity for long-term survival following treatment for carcinomatosis requires a complete cytoreduction in all reports for gastrointestinal cancer. Quantitative prognostic indicators need to be knowledgeably employed when patients with carcinomatosis are being treated. Improved patient selection with greater benefit and reduced morbidity and mortality should result.

## I. Introduction

Peritoneal carcinomatosis has always been regarded as a terminal condition. It is present in 10 to 30% of patients with gastrointestinal cancer at the time of their initial surgery and is a frequent finding in patients who develop recurrent cancer. Important natural history studies establish a 6-month median survival in this group of patients [1-3]. Recent multicenter phase II and a single phase III study evaluating the usefulness of cytoreductive surgery and perioperative intraperitoneal chemotherapy are promising [4,5]. Patient selection is of utmost importance in optimizing the results of treatment and excluding patients who will not benefit from a high morbidity and potentially life threatening therapy.

Quantitative prognostic indicators are to serve as guidelines in the selection of treatments to maximize benefits of therapy and to exclude patients who have little or no chance to improve. They are of greatest utility in high risk and costly management protocols. Requirements of a useful quantitative prognostic indicator include reproducibility, prediction of survivorship, and assessment of morbidity and mortality. The goal is to establish management protocols that standardize the decision making process for multiple caregivers.

General surgery has used quantitative prognostic indicators in the past with established benefit to patient care. Examples of quantitative prognostic indicators currently in use include Ranson's criteria, which estimates the risk of life threatening complication or death in patients with acute pancreatitis; and, the Child-Pugh score for liver cirrhosis, which evaluates the severity of liver disease correlating grades with one- and two-year survival. Currently, there are several clinical assessments at many different institutions in use for the evaluation of carcinomatosis (see Table 1). Our goal in this manuscript is to critically discuss these quantitative prognostic indicators. Collaborative studies between institutions would be greatly facilitated with standardized clinical tools for management of carcinomatosis from gastrointestinal cancer.

**Table 1:** Quantitative prognostic indicators currently in use in patients with carcinomatosis.

Tumor histopathology
Intraoperative assessment of the extent of carcinomatosis at time of surgical exploration
• Gilly peritoneal carcinomatosis staging
• Carcinomatosis staging by the Japanese Research Society for Gastric Cancer
• Peritoneal Cancer Index (PCI)
• Dutch Simplified Peritoneal Carcinomatosis Index (SPCI)

CT PCI

Prior Surgical Score

Completeness of Cytoreduction Score

## II. Histopathology

In patients with carcinomatosis from gastrointestinal cancer, invasive implants are disseminated within the peritoneal cavity. However, in two conditions the biological aggressiveness of the disease will have a broad spectrum. These two diseases are mucinous appendiceal malignancies (oftentimes clinically designated pseudomyxoma peritonei syndrome) and peritoneal mesothelioma. In these diseases a non-invasive process may be widely disseminated on the peritoneal surfaces. The biological aggressiveness of the malignancy can be estimated by the pathologist in a knowledgeable histologic review of multiple specimens. For pseudomyxoma peritonei syndrome, the histologic classification described by Ronnett and colleagues has been most widely utilized [6].

Histopathologic examination categorizes the disease process into disseminated peritoneal adenomucinosis (DPAM), peritoneal mucinous carcinoma (PMCA) or a hybrid type.

### Disseminated Peritoneal Adenomucinosis

**Figure 1:**
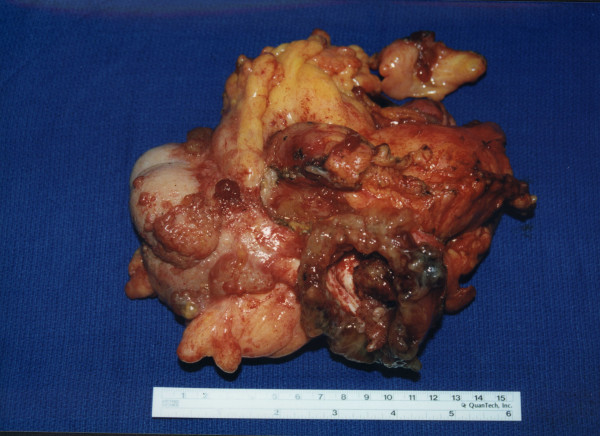
Right colon, terminal ileum and mucocele of the appendix. This appendix is greatly dilated; the end has ruptured releasing mucus and adenomatous epithelial cells into the free peritoneal cavity.

**Figure 2:**
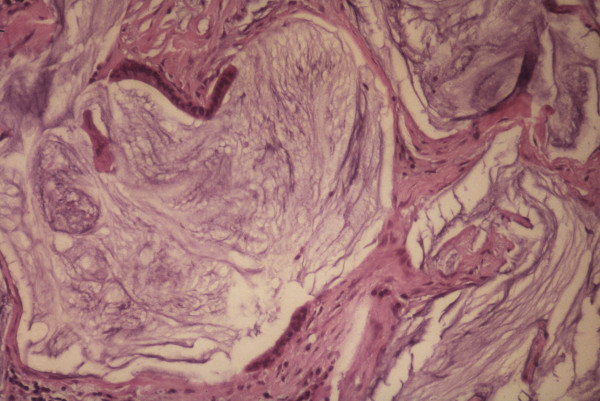
Histopathology of disseminated peritoneal adenomucinosis (DPAM). (H+E × 200)

This is a minimally invasive disease, and therefore more likely to be completely removed by cytoreduction using peritonectomy. The histology of DPAM shows a bland single layer of epithelium that surrounds lobules of mucin. There are no signet rings and there is minimal atypia. Invasion of the structures upon which tumor accumulates does not occur. The primary site for DPAM is an appendiceal adenoma which has minimally invaded the wall of the appendix. Usually, the widespread intraperitoneal dissemination of mucinous tumor is caused by a rupture of the lumen of the appendix from pressure built up by the malignant mucocele. Figure 1 shows the typical disruption of the wall of the appendix by tumor. Figure 2 presents the histologic character of DPAM.

### Peritoneal Mucinous Adenocarcinoma

**Figure 3:**
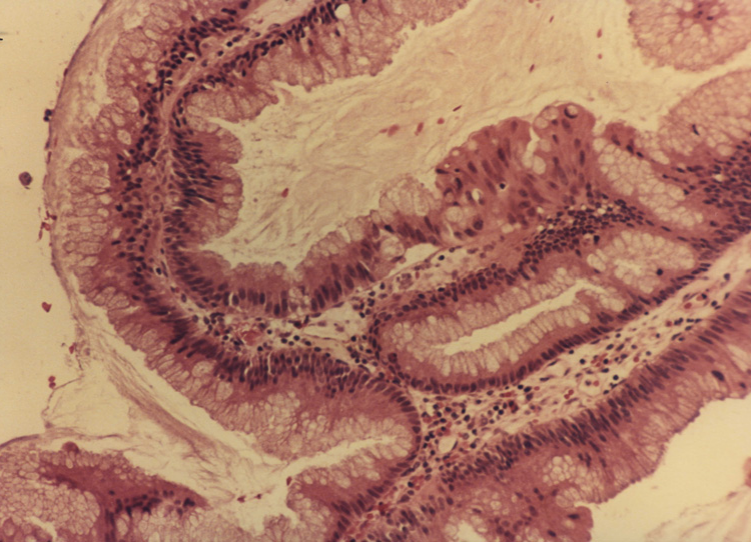
Histopathology of peritoneal mucinous adenomucinosis (PMCA). (H+E × 700)

This is an invasive disease in which the mucinous cancer cells show invasion into surrounding tissues. Sometimes, the signet ring morphology or lymph node metastases are present. The cancer cells will be found in multiple layers surrounding the mucinous tumor globules. There is loss of nuclear polarity and atypia is common. The quantity of mucus may be variable from one patient to the other. However, the PMCA histology may be associated with very large amounts of mucoid ascites fluid. Therefore, it is categorized as pseudomyxoma peritonei syndrome but with an aggressive tumor histology. Figure 3 provides an example of PMCA.

### Hybrid Type Disease

**Figure 4:**
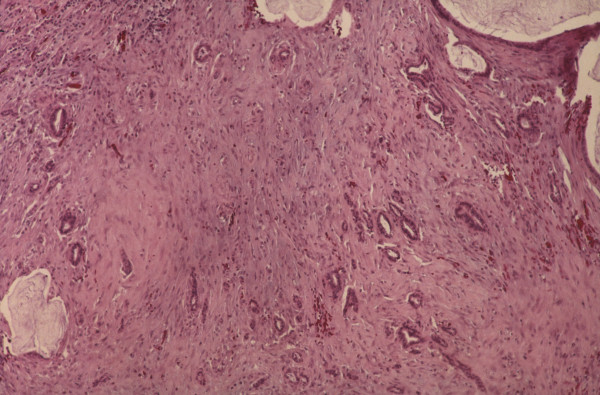
Histopathology of hybrid type mucinous appendiceal malignancy. (H+E × 100)

In the hybrid type of mucinous carcinomatosis the field of view for the pathologist shows 95% or more DPAM. PMCA is present but in 5% or less of the total field of view (figure 4). If there is more than 5% PMCA the histology is no longer hybrid type but designated as PMCA.

Not surprisingly, the observation has been made by numerous groups that the non-invasive mucinous tumors (DPAM and hybrid type) are amenable to complete cytoreduction. Therefore more definitive treatment and improved survival using the combined approach is expected with DPAM and hybrid type [7]. The histologic and clinical differences between the different types of mucinous appendiceal and other gastrointestinal mucinous tumors are shown in Table 2.

**Table 2:** Histopathologic features of epithelial mucinous tumors of appendiceal, colonic, and small bowel origin are designated as disseminated peritoneal adenomucinosis (DPAM) and peritoneal mucinous carcinomatosis (PMCA).

**Features**	**DPAM**	**PMCA**
Primary site	Appendix	Appendix, colon, small intestine
Primary diagnosis	Mucinous adenoma usually in a mucocoele	Mucinous adenocarcinoma
Surgical appearance	Mucinous tumors and mucinous ascites with redistribution	Carcinomatosis with variable amounts of mucinous ascites, redistribution is prominent with large volume of ascites
Peritoneal tumor		
• Cellularity	Scant	Moderate to abundant
• Morphology	Abundant extracellular mucin containing simple to focally proliferative mucinous epithelium. There is a single layer of cells	Moderate to abundant extracellular mucin containing extensively proliferative mucinous epithelium or mucinous glands, clusters of cells, or individual cells consistent with carcinoma
• Cytologic atypia	Minimal	Moderate to marked
• Mitotic activity	Rare	Infrequent to frequent

Lymph node involvement	Almost never	Moderate

Liver metastases	Almost never	Very infrequent

Parenchymal organ invasion	Rare (except ovary)	Frequent

Hybrid type tumors show less than 5% of PMCA within DPAM. Mucinous carcinomas are divided into three grades by maintenance or loss of glandular architecture.

## III. Intraperitoneal Assessment of the extent of Carcinomatosis

The quantitation of tumor found at the time of surgical exploration of the abdomen has proven to be of value in assessment of prognosis and treatment planning. Four different assessments have been published. They are listed in Table 1.

### Gilly Peritoneal Carcinomatosis Staging

The Gilly peritoneal carcinomatosis staging format was first described in Lyon in 1994 [8]. This prognostic tool takes into account the size of lesions found at operation (table 3). Two advantages of this system are simplicity and reproducibility. The utility of the Gilly staging device in survivorship prediction has been demonstrated in the multicentric prospective EVOCAPE study which gathered data from 370 patients with peritoneal carcinomatosis from non-gynecologic malignancies [2]. A significant difference was observed between stages 1 and 2 with a median survival of 6 months and stages 3 and 4 whose median survival was 3 months. The Gilly carcinomatosis staging has also been validated in patients having combined treatment for carcinomatosis [9].

**Table 3:** Gilly peritoneal carcinomatosis staging.

**Stage**	**Peritoneal carcinomatosis description**
Stage 0	No macroscopic disease
Stage 1	Malignant implants less than 5 mm in diameter Localized in one part of the abdomen
Stage 2	Diffuse to the whole abdomen
Stage 3	Malignant implants 5 mm to 2 cm
Stage 4	Large malignant nodules (more than 2 cm)

Although the Gilly system has been used for almost a decade with acceptable prognostic value, there are some criticisms regarding this system. First, it should not be designated a "staging system" because patients can only be staged once in the course of their disease at the time of diagnosis of the primary malignancy. Usually, a TNM staging system is appropriate. The system might better be called the Gilly prognostic index for carcinomatosis.

A second weakness of the Gilly prognostic index concerns a failure to quantitate distribution of peritoneal surface implants in the stage 3 and 4 categories. Carcinomatosis confined to one portion of the abdomen may carry an excellent prognosis even if the localized tumor implants are of large size. If group III and group IV nodules by size are diffuse throughout the whole abdomen, certainly a much different prognosis would occur. A definitive assessment of not only the size of the nodules but also the distribution of carcinomatosis is necessary for the most accurate assessment of prognosis.

The Japanese have proposed a quantitation of carcinomatosis that is very simple, has been frequently applied, and has been validated for gastric malignancy. For the original staging a "P factor" is indicated for gastric cancer patients. P-0 means that no carcinomatosis was seen by the surgeon or could be established at the time of surgery. It would currently include patients who are cytology positive for gastric cancer cells. P-1 indicates implants immediately adjacent to the stomach and above the transverse colon. P-2 indicates scattered implants within the abdomen but not of great number. P-3 indicates numerous implants throughout the abdomen and pelvis.

This staging system can also be applied to patients who have carcinomatosis with recurrent gastric cancer. A major deficit of this staging system is its inability to accurately locate the carcinomatosis. Also, it has no size assessment of the cancerous implants. Although the P factor has been of great value historically in the management of primary gastric cancer as peritonectomy and intraperitoneal chemotherapy are used for treatment of carcinomatosis, a more precise prognostic assessment is needed to manage gastric cancer peritoneal seeding.

### Peritoneal Cancer Index

The Peritoneal Cancer Index (PCI), like the other carcinomatosis assessments, is determined at the time of surgical exploration of the abdomen and pelvis. With invasive cancer it serves as an estimate of probability of complete cytoreduction and has been found to be an accurate assessment of survival when cytoreductive surgery and perioperative intraperitoneal chemotherapy are used as treatment [10].

**Figure 5:**
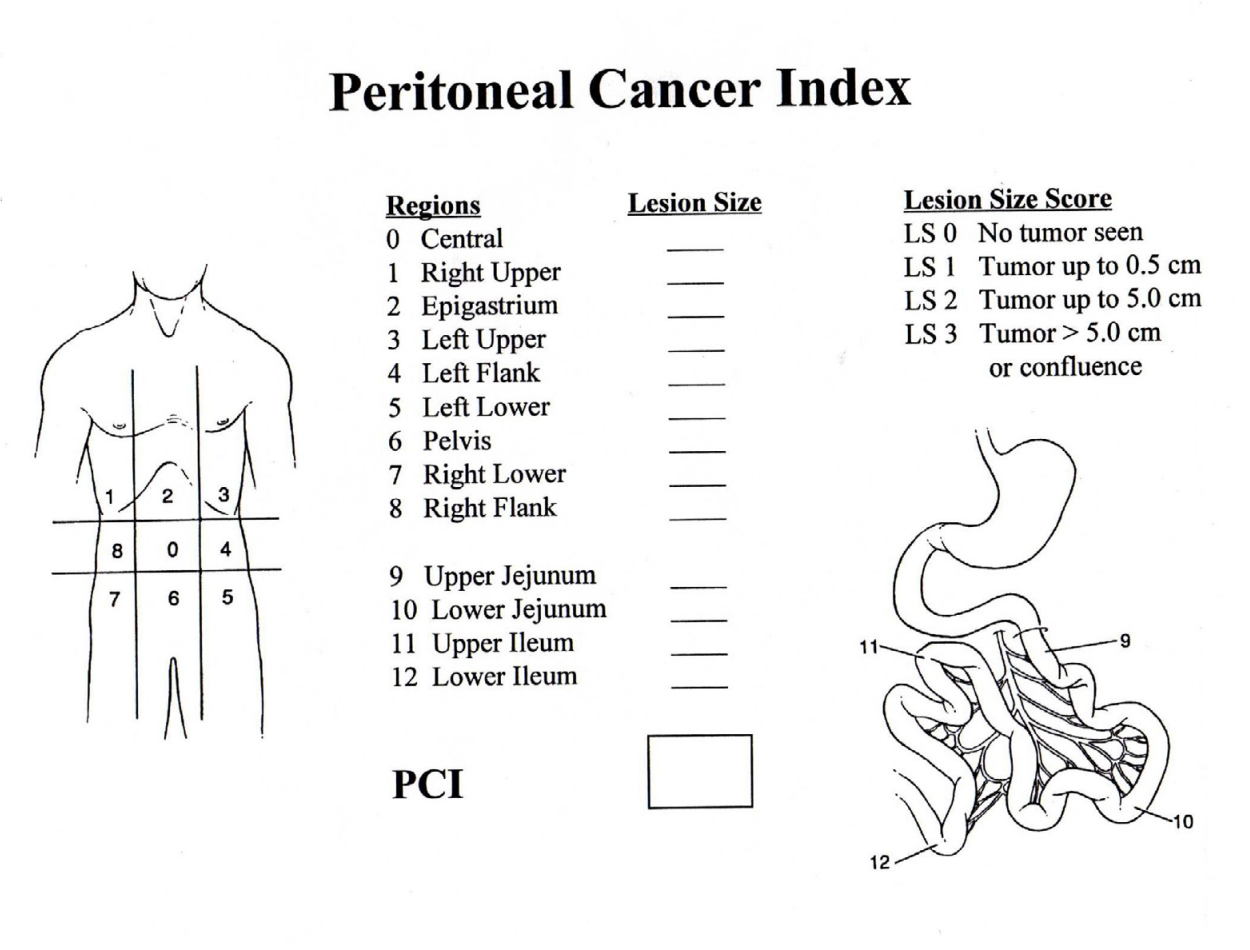
Peritoneal cancer index (PCI). Two transverse planes and two sagittal planes divide the abdomen into 9 regions. The upper transverse plane is located at the lowest aspect of the costal margin and the lower transverse plane is placed at the anterior superior iliac spine. The sagittal planes divide the abdomen into three equal sectors. The lines define the nine regions which are numbered in a clockwise direction with 0 at the umbilicus and 1 defining the space beneath the right hemidiaphragm. Regions 9–12 divide the small bowel. Lesion size score is determined after complete lysis of all adhesions and the complete inspection of all parietal and visceral peritoneal surfaces. It refers to the greatest diameter of tumor implants that are distributed on the peritoneal surfaces. Primary tumors or localized recurrences at the primary site that can be removed definitively are excluded from the lesion size assessment. If there is confluence of disease matting abdominal or pelvic structures together, this is automatically scored as L-3 even if it is a thin confluence of cancerous implants.

The PCI quantitatively combines the distribution of tumor throughout 13 abdominopelvic regions with a lesion size score. Two transverse and two sagittal planes divide the abdomen into 9 regions. The upper transverse plane is located at the lowest aspect of the costal margin, and the lower transverse plane is placed at the anterior superior iliac spine. The sagittal planes divide the abdomen into three equal sectors. The lines define 9 regions, which are numbered in a clockwise direction with 0 at the umbilicus and 1 defining the space beneath the right hemidiaphragm. Regions 9 through 12 divide the small bowel into upper and lower jejunum and upper and lower ileum (Figure 5). To make the PCI tool more quantitative and reproducible, each region is not only defined by the surface landmarks as previously described, but can also be defined by the anatomic structures found in each region (Table 4).

**Table 4:** Anatomic structures involved in the 13 abdominopelvic regions of the peritoneal cancer index (PCI).

**Regions**	**Anatomic structures**
0 Central	Midline abdominal incision – entire greater omentum – transverse colon
1 Right upper	Superior surface of the right lobe of the liver – undersurface of the right hemidiaphragm – right retro hepatic space
2 Epigastrium	Epigastric fat pad – left lobe of the liver – lesser omentum – falciform ligament
3 Left upper	Undersurface of the left hemidiaphragm – spleen – tail of pancreas – anterior and posterior surfaces of the stomach
4 Left flank	Descending colon – left abdominal gutter
5 Left lower	Pelvic sidewall lateral to the sigmoid colon – sigmoid colon
6 Pelvis	Female internal genitalia with ovaries, tubes and uterus – bladder, Douglas pouch – rectosigmoid colon
7 Right lower	Right pelvic sidewall – cecum – appendix
8 Right flank	Right abdominal gutter – ascending colon
9 Upper jejunum	
10 Lower jejunum	
11 Upper ileum	
12 Lower ileum	

The lesion size (LS) score is determined after complete lysis of all adhesions and complete inspection of all parietal and visceral peritonea surfaces within the abdominopelvic regions. LS-0 indicates no implants seen. LS-1 indicates implants less than 0.25 cm. LS-2 indicates implants between 0.25 and 2.5 cm. LS-3 indicates implants greater than 2.5 cm. It refers to the greatest diameter of tumor implants that are distributed on the peritoneal surfaces. Primary tumors or localized recurrences at the primary site that can be removed definitively are excluded from the assessment. If there is a confluence of disease matting abdominal or pelvic structures together, this is automatically scored as LS-3 even if it is a thin layer of cancerous implants.

The lesion sizes are then summated for all abdominopelvic regions. The extent of the disease within all regions of the abdomen and pelvis is indicated by a numerical score from 0 to 39.

**Figure 6:**
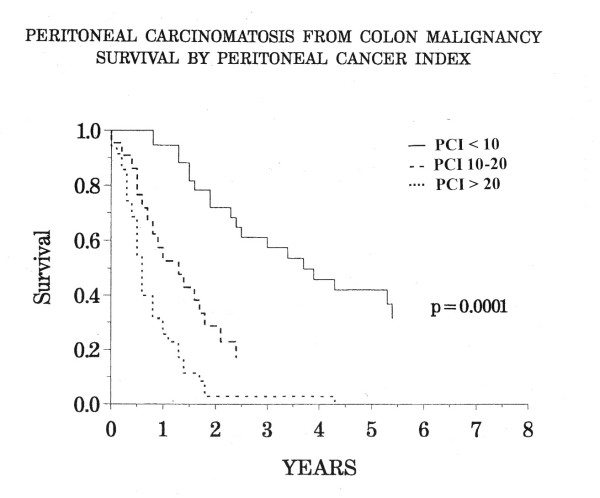
Peritoneal carcinomatosis from colon malignancy survival by peritoneal cancer index. (Modified from Reference 13)

In 1995, Sugarbaker and Jablonski published that the PCI was a meaningful assessment for colon cancer but not for mucinous appendiceal tumors [11]. Elias et al., found survival to be more favorable in those patients with carcinomatosis from colon cancer with a PCI score of less than 16 [12]. In a larger number of patients Sugarbaker and Chang established survivorship using the PCI [13]. Five-year survival was 50% in colon cancer patients with carcinomatosis with a PCI less than 10, 20% for 11–20 and 0% in those with a PCI score greater than 20 (Figure 6). Tentes and colleagues validated the PCI for ovarian cancer [14]. The PCI is not only useful as a prognostic indicator but also as a guide for sequential determinations of volume of carcinomatosis over time estimating the likelihood of a complete cytoreduction at re-operative surgery [15].

This quantitative prognostic indicator for colon carcinomatosis established that for patients scoring greater than 20, palliation is the goal of treatment. Currently, a PCI of greater than 20 is regarded as a relative contraindication to an elective intervention for carcinomatosis from colon cancer. It is associated with a low median survival, approximately the same as median survival without surgical intervention. In patients who have a PCI greater than 20, palliative surgery is indicated in order to alleviate symptoms or to prevent symptoms that may occur in the near future. In an asymptomatic patient with colon carcinomatosis cytoreductive surgery with intraperitoneal chemotherapy with cure as a goal of treatment is probably not indicated.

An exception to the utility of the PCI is found in treating patients with pseudomyxoma peritonei and minimally aggressive mesothelioma. Because the disease is non-invasive, a PCI of 39 can be converted to 0 by cytoreductive surgery. There is a low probability of recurrence after complete cytoreduction with perioperative intraperitoneal chemotherapy and therefore the PCI has no prognostic implication [7].

Another caveat that must be observed when using the PCI occurs in cases in which a low PCI score is recorded in the presence of invasive cancer at a crucial anatomic site. For example, at exploration one may find invasive tumor in and around the common bile duct with little disease elsewhere. Even thought the PCI is low, a complete cytoreduction may not be possible. In these cases, invasive cancer at a crucial anatomic site places the patient into the same category as would systemic metastasis in the lungs or bone. Only palliative surgery is indicated if residual disease post-cytoreduction will be present.

### Simplified Peritoneal Cancer Index

The Simplified Peritoneal Cancer Index (SPCI) was established at the Netherlands Cancer Institute and has been used for colorectal and appendieal cancer staging (Table 5). This tool has prognostic implication for survival following cytoreductive surgery and hyperthermic intraperitoneal chemotherapy [16].

**Table 5:** Simplified Peritoneal Cancer Index

◆ Tumor is recorded as:
indent="1" • Large (> 5 cm)
indent="1" • Moderate (1–5 cm)
indent="1" • Small (< 1 cm)
indent="1" • None
◆ Seven abdominal regions:
indent="1" • I: pelvis
indent="1" • II: right lower abdomen
indent="1" • III: greater omentum, transverse colon and spleen
indent="1" • IV: right subdiaphragmatic area
indent="1" • V: left subdiaphragmatic area
indent="1" • VI: subhepatic and lesser omental area
indent="1" • VII: small bowel and small bowel mesentery

Verwaal and colleagues have provided important information regarding the relationship of the Simplified Peritoneal Cancer Index and the incidence of complications in patients who receive combined treatment [17]. In their review of the toxicity of combined treatment, complications increased when the cancer index recorded involvement of more than five regions (p = 0.044). Also, if the patient had recurrent colon cancer (as opposed to carcinomatosis with primary cancer) or if there was an incomplete cytoreduction, the incidence of complications was significantly higher. Verwaal et al., established that the peritoneal cancer index quantitated not only the survival outcome of these patients but also the expected morbidity and mortality of the combined treatment [16].

There are marked similarities between the SPCI and the PCI. Both the anatomic distribution of the tumor masses and the size of the tumor masses within each abdominal region are indicated. In the PCI, there are 13 anatomic sites designated by a diagram; in the Dutch SPCI, there are 7 anatomic regions designated by anatomic site. In both systems the volume of tumor in each region is to be scored quantitatively. Some shortcomings of the SPCI could be formulated. First, the epigastric region, very important in determining the completeness of cytoreduction in some diseases is not designated separately. Disease above the stomach in the lesser omental region may cause the cytoreduction to be incomplete [15].

A second major criticism of the Dutch SPCI concerns their misuse of their own tool. In their recent publications they perform a survival analysis by SPCI and a toxicity assessment by the SPCI. However, only the involvement of regions 0–7 was indicated. No tumor size in the regions was indicated [16,17].

## Prior Surgical Score

An accepted fact regarding cancer treatment is that the optimal treatment with the highest cure rate, the greatest preservation of function, and the lowest morbidity and mortality is the initial treatment. In the management of carcinomatosis the extent of prior resection before definitive cytoreduction with intraperitoneal chemotherapy has a negative impact on the survival. This occurs because of the cancer cell entrapment phenomenon. Surgery opens tissue planes whose raw surface is a favored site for cancer cell adherence, vascularization and progression. In the use of combined treatment for carcinomatosis, the non-traumatized peritoneal surface is the body's first line of defense against carcinomatosis. Cancer progression deep to peritoneal surfaces, especially disease imbedded in scar, is difficult or impossible to remove by peritonectomy or to eradicate by intraperitoneal chemotherapy.

The prior surgical score (PSS) quantitates the extent of surgery prior to definitive combined treatment. It shows that the greater the surgery the poorer the results of carcinomatosis treatment. The assessment uses a diagram similar to that for PCI but excludes abdominopelvic regions 9–12. For a PSS of 0 no prior surgery or only a biopsy was performed; PSS of 1 indicates one region with prior surgery; PSS-2 indicates 2 to 5 regions previously dissected; PSS-3 indicates more than 5 regions previously dissected. This is equivalent to a prior attempt at complete cytoreduction but in the absence of perioperative intraperitoneal chemotherapy. In appendiceal cancer patients with a prior surgical score of 0–2, the survival using combined treatment was 70% at 5 years; with a prior surgical score of 3, the 5-year survival was 51% (p = 0.001) [18].

## Completeness of Cytoreduction Score

The Completeness of Cytoreduction Score functions as a major prognostic indicator for the survival in peritoneal mesothelima, colon cancer with carcinomatosis, gastric cancer with carcinomatosis and sarcomatosis [7]. It is to be assessed after cytoreductive surgery is completed. Complete cytoreduction (CC-0 or CC-1) or incomplete (CC-2 or CC-3) are determined. A CC-0 is apparent when there is no peritoneal seeding visualized within the operative field. CC-1 indicates nodules persisting after cytoreduction less than 2.5 cm. CC-2 has nodules between 2.5 and 5 cm, whereas a CC-3 indicates nodules greater than 5 cm or a confluence of unresectable tumor nodule at any site within the abdomen or pelvis. The CC-1 tumor nodule size is thought to be penetrable by intracavitary chemotherapy and is, therefore, designated as complete cytoreduction if perioperative intraperitoneal chemotherapy is used.

**Figure 7:**
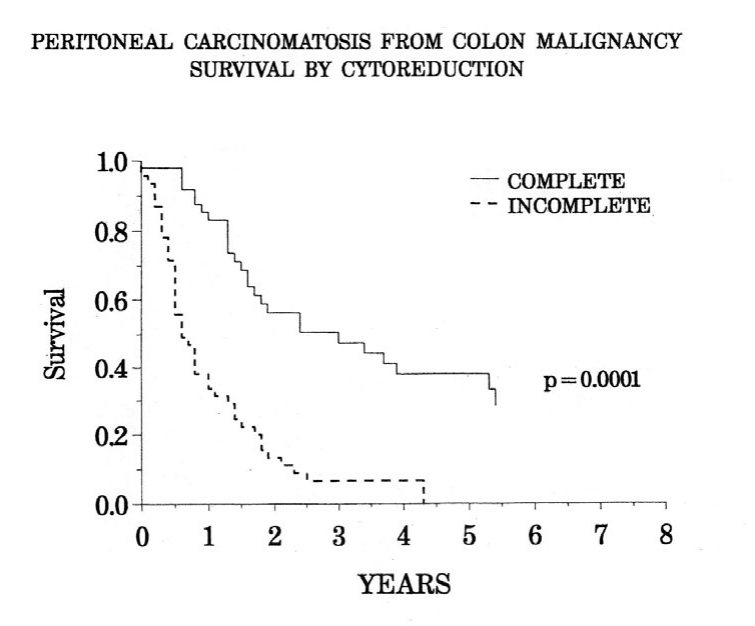
Peritoneal carcinomatosis from colon malignancy survival by cytoreduction. (Modified from Reference 13)

Sugarbaker and colleagues found that prognosis can be estimated by completeness of cytoreduction. For colon cancer as shown in Figure 7, there is a 40% chance of survival at 5 years in those who undergo complete cytoreduction versus 0% survival in the incomplete category [7,13]. Numerous other groups have confirmed the complete cytoreduction as a requirement for survival after treatment of carcinomatosis from appendiceal, colorectal and gastric cancer [4,9,11,12,16-19].

Although no formal statement in the literature is available, it is thought that the definition of complete vs. incomplete cytoreduction varies with the histologic type of the malignancy. For example, mucinous tumors by diffusion are well penetrated with intraperitoneal chemotherapy solutions. With minimally invasive mucinous tumors such as pseudomyxoma peritonei, complete cytoreduction may occur in the combined treatment plan with tumor nodules up to a full centimeter in size. In contrast, hard fibrotic non-mucinous colon cancer is poorly penetrated by chemotherapy solution. Only cytoreduction down to no visible evidence of disease would be expected to result in long-term survival with a sclerotic malignant process. Also, some cancers may be remarkably more responsive to chemotherapy than others. This is likely the case with a majority of ovarian cancers. Their complete response to systemic chemotherapy is also frequently seen with intraperitoneal chemotherapy solutions or a bidirectional (intraperitoneal combined with intravenous chemotherapy) approach. In both these situations the definition of a complete cytoreduction scored by a CC-1 designation would vary with the clinical situation.

## Computerized Tomographic PCI

The preoperative CT is an excellent tool in locating and quantifying mucinous adenocarcinoma within the peritoneal cavity [20]. Unfortunately, with intestinal histologic type of colon cancer the accuracy of the CT is considerably reduced [21]. However, for mucinous carcinomatosis CT scanning is an accurate prognostic indicator of the possibility of resectability. It may show segmental obstruction of the small bowel or tumor nodules greater than 5 cm on small bowel. Patients who have both of these findings have a likelihood of less than 5% of complete cytoreduction. Obstructed segments of bowel signal an invasive character of malignancy on small bowl surfaces that would be unlikely to be completely cytoreduced. Large tumor nodules on small bowel or its mesentery are unlikely to be adequately cytoreduced without visceral resection.

There are some special demands on CT scanning if the radiologic examination is to be optimized. Bowel loops cut in cross section are often indistinguishable from cancer nodules. Only if maximal oral contrast using a barium sulfate compound is utilized to prepare the patient for this examination can the greatest accuracy and the greatest prognostic implications of the examination be realized.

Another technical requirement is the imaging of solid tumor layered out on the peritoneal surfaces. Unless there is maximal intravenous contrast with a 60 to 120 second delay after contrast infusion will the confluence of malignancy as a thin layer on the peritoneum be imaged. In some patients, the solid tumor, or semisolid tumor may be distributed to appear as ascites on abdominal and pelvic CT. Much to the surgeon's dismay, upon opening the abdomen, a solid tumor mass filling the abdomen and pelvis and causing adherence of small bowel and small bowel mesentery will be revealed. In this situation, not even palliative surgery can be safely performed. In patients who clinically have a firm abdomen and in whom the surgeon suspects large volume of solid tumor, an ultrasound examination may be required in order to confirm an ascitic versus a solid component of the abdominal and pelvic malignancy. If ultrasound shows that there is only minimal or no ascites and that the large volume of tumor is solid or semisolid, surgical interventions are not beneficial. It is better to determine the nature of the carcinomatosis radiologically than at the time of a major surgical exploration.

## Conclusion

Quantitative prognostic indicators are of value in management of peritoneal surface malignancy from gastrointestinal cancer. Preoperative CT PCI, intraoperative PCI and postresection CC score have all been reported valuable. As one knowledgeable applies these tests, proper selection of patients for combined treatment may increase benefit and decrease morbidity and mortality.
